# Regulation of ATP levels in *Escherichia coli* using CRISPR interference for enhanced pinocembrin production

**DOI:** 10.1186/s12934-018-0995-7

**Published:** 2018-09-18

**Authors:** Sha Tao, Ying Qian, Xin Wang, Weijia Cao, Weichao Ma, Kequan Chen, Pingkai Ouyang

**Affiliations:** State Key Laboratory of Materials-Oriented Chemical Engineering, College of Biotechnology and Pharmaceutical Engineering, Nanjing Tech University, Nanjing, 211816 Jiangsu China

**Keywords:** Flavonoids, ATP, CRISPRi, Pinocembrin

## Abstract

**Background:**

Microbial biosynthesis of natural products holds promise for preclinical studies and treating diseases. For instance, pinocembrin is a natural flavonoid with important pharmacologic characteristics and is widely used in preclinical studies. However, high yield of natural products production is often limited by the intracellular cofactor level, including adenosine triphosphate (ATP). To address this challenge, tailored modification of ATP concentration in *Escherichia coli* was applied in efficient pinocembrin production.

**Results:**

In the present study, a clustered regularly interspaced short palindromic repeats (CRISPR) interference system was performed for screening several ATP-related candidate genes, where *met*K and *pro*B showed its potential to improve ATP level and increased pinocembrin production. Subsequently, the repression efficiency of *met*K and *pro*B were optimized to achieve the appropriate levels of ATP and enhancing the pinocembrin production, which allowed the pinocembrin titer increased to 102.02 mg/L. Coupled with the malonyl-CoA engineering and optimization of culture and induction condition, a final pinocembrin titer of 165.31 mg/L was achieved, which is 10.2-fold higher than control strains.

**Conclusions:**

Our results introduce a strategy to approach the efficient biosynthesis of pinocembrin via ATP level strengthen using CRISPR interference. Furthermore coupled with the malonyl-CoA engineering and induction condition have been optimized for pinocembrin production. The results and engineering strategies demonstrated here would hold promise for the ATP level improvement of other flavonoids by CRISPRi system, thereby facilitating other flavonoids production.

**Electronic supplementary material:**

The online version of this article (10.1186/s12934-018-0995-7) contains supplementary material, which is available to authorized users.

## Background

**Fig. 1:**
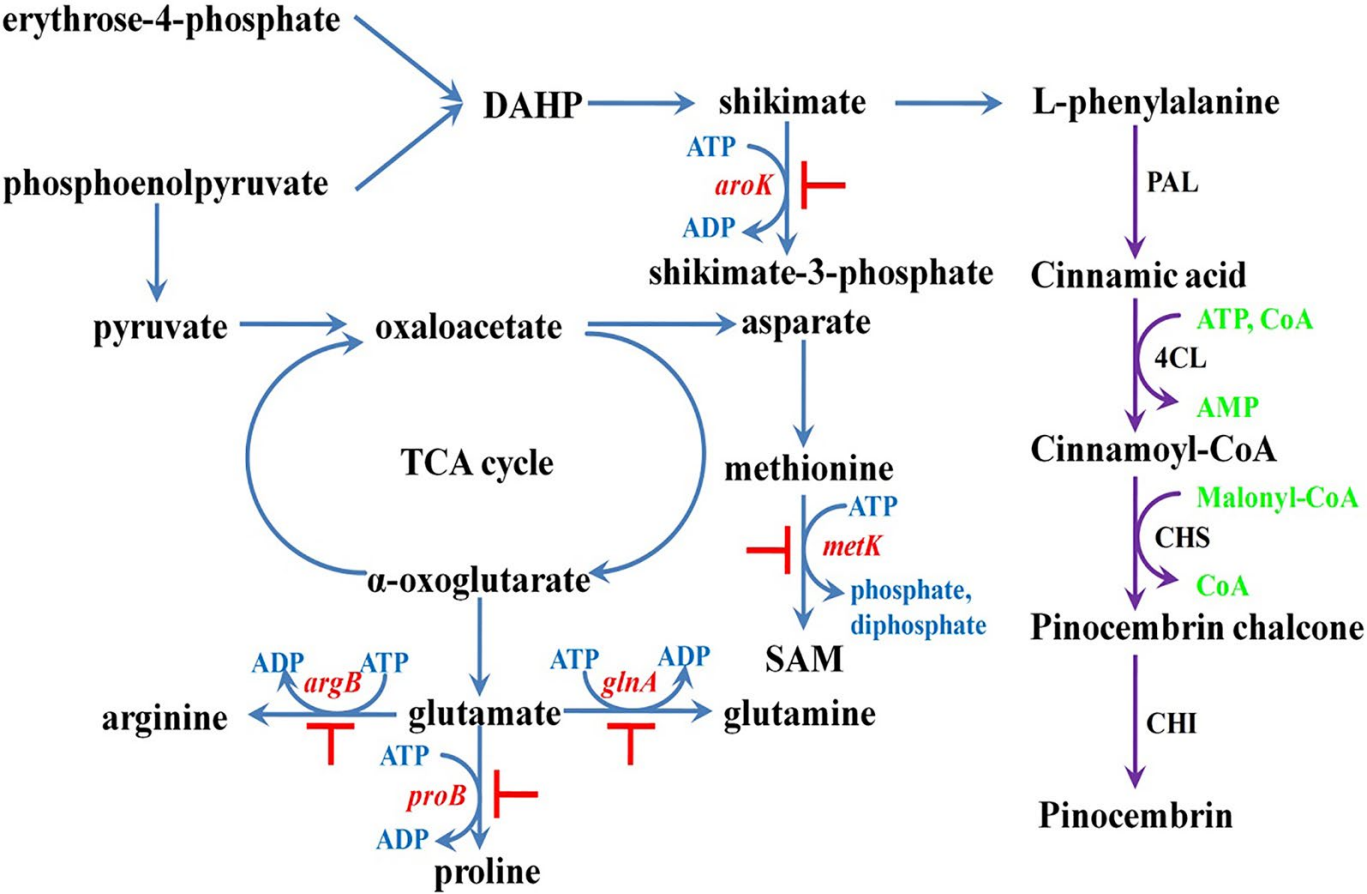
Metabolic pathway for pinocembrin production in *E. coli*. In the metabolic map, reactions from the pinocembrin pathway are shown in violet and Up-regulations are shown blue. DAHP, 3-deoxy-d-arabino-heptulosonate-7-phosphate; SAM, S-adenosylmethionine; PAL, phenylalanine ammonia lyase encoded by *pal*; 4CL, 4-coumaroyl-CoA ligase encoded by *4cl*; CHS, chalcone synthase encoded by *chs*; CHI, chalcone isomerase encoded by *chi*

The natural products from higher plants include various chemicals, such as alkaloids, isoprenoids and phenolic compounds (phenylpropanoids and flavonoids) [1]. Although most natural products were obtained from plants, the efficient production of natural products is limited by the strict control of secondary metabolism in plants [2]. In recent years, with the advances of the synthetic biology, the microbial production emerged as an attractive approach for the discovery and production of natural products [3]. Pinocembrin is a natural flavonoid with important pharmacologic characteristics and is widely used in preclinical studies [4, 5]. The microbial biosynthesis of pinocembrin showed a great potential for the cost-efficient production and large-scale industrialization [6]. The bio-strategy to produce pinocembrin could be accomplished by assembling of phenylalanine ammonia-lyase (PAL); 4-coumarate-CoA ligase (4CL); chalcone synthase (CHS) and chalconeisomerase (CHI) [5]. Biosynthetic pathway of pinocembrin involves three cofactors (ATP, CoA, malonyl-CoA) as precursors (Fig. 1).

The biosynthetic pathways of natural products composed of enzymes with several protein subunits are active only in the presence of energy carriers (ATP) [7]. For example, among the 971 reactions of natural product biosynthesis identified in *Streptomyces. coelicolor*, more than 21% rely on the participation of ATP [8]. Also, ATP is required for reconstitution of heterocycle forming activity with the microcin B17 precursor [9]. Previous research suggested that, ATP pathway modification might be an alternative way to enhance the final product biosynthesis. For instance, Zhao found that the increase of ATP supply would improve the production of terpenoids compounds [10].

ATP is synthesized through ATP synthase and electron transfer chain in *E. coli* [10]. In order to increase ATP supply, different gene operons were modulated in *E. coli* with five regulatory parts, lead to 20%, 16%, 5% and 21% increase in β-carotene yield [10]. In addition, the additive of sodium citrate acts as an auxiliary energy substrate for the accumulation of intracellular ATP and the increase of ATP/ADP ratio, lead to the overproduction of *S*-adenosylmethionine (SAM) and glutathione (GSH) [11]. Although the overexpression of ATPase and optimization of fermentation process are effective ways to improve ATP supply, the repression of the consumption pathway to increase the ATP accumulation is an alternative option [12]. It was reported that the disruption of the ATP consuming bypass pathway in *Saccharomyces cerevisiae* could achieve 3.1-fold higher ATP-generating activity and 1.7-fold higher glutathione productivity compared with the control strain. Furthermore, Chen [13] found that the sRNA-based downregulation strategy in *E. coli* could increase the ATP level and S-adenosylmethionine production. Recently, the clustered regularly interspaced short palindromic repeats interference (CRISPRi) system provides an efficient tool for targeted gene regulation in a sequence-specific manner in *E. coli* [14]. Compared with sRNAs and antisense RNAs strategies [15, 16], this RNA-guided DNA recognition platform provides a simple approach for repression of multiple target genes simultaneously on a genome-wide scale [17–19]. Accordingly, the development of genetic engineering has offered an alternative approach utilizing a CRISPRi regulation strategy to control the intracellular ATP level. In the biosynthesis of flavonoid, ATP has been proved not only to provide adenosyl moiety in *E. coli* metabolism but also to play the role of an energy carrier in pinocembrin biosynthesis. However, there is no ATP engineering strategies for increasing pinocembrin biosynthesis has been proposed to date.

In this study, we demonstrated that the ATP additive was advantageous for pinocembrin production and 5 genes involved in ATP metabolic pathways were repressed by the CRISPRi system to identify individual target genes that could increase the ATP level and pinocembrin production. Furthermore, multiple genes repressing for ATP engineering was performed to achieve the most effective combination. Finally, the pinocembrin production was further improved by combination of ATP and malonyl-CoA engineering. The results and engineering strategies demonstrated here would provide an alternative platform to improve intracellular ATP level in the biosynthesis pathway of natural products.

## Methods

### Strains and media

*Escherichia coli* Trans-T1 was used for plasmid propagation. *E. coli* BL21 (DE3) was used to express sgRNA and produce pinocembrin. The compatible vector set (pTrc99a, pCDF303, pRSFDuet-1, and pACYCDuet-1) was used to express multiple genes in one strain. *E. coli* seed cultures were grown in Luria–Bertani (LB) medium. MOPS minimal medium supplemented with 5 g/L glucose and 4 g/L NH_4_Cl was used for sgRNA expression and pinocembrin production. MOPS minimal medium contains 10× MOPS mixture 100 mL, 0.132 M K_2_HPO_4_ 10 mL, milliQ H_2_O 880 mL, 1 mg/mL thiamine 0.1 mL. Appropriate antibiotics were added at the following concentrations: 100 μg/mL of ampicillin, 50 μg/mL of kanamycin, 34 μg/mL chloramphenicol and 40 μg/mL streptomycin. Cell growth was detected via the optical density measured at 600 nm (OD_600_) using a spectrophotometer (Thermo Scientific, Waltham, MA, USA). The plasmids and strains used in this study are listed in Tables 1 and 2.

**Table 1:** Plasmids used in this study

Plasmid	Plasmid description	Source
44249-dcas9	P15A Replicon Cm^R^	Addgene
pACYC-dcas9	The dCas9 gene on plasmid 44249-dCas9 was inserted into the BglII and XhoI restriction sites of the pACYCDuet-1 plasmid	This study
pCDF303	Trc promoter, CloE1 replicon, Sm^R^	This study
pCDF303-*gfp*-H	Plasmid is used for high intensity repression of *gfp*	This study
pCDF303-*gfp*-M	Plasmid is used for medium intensity repression of *gfp*	This study
pCDF303-*gfp*-L	Plasmid is used for low intensity repression of *gfp*	This study
pCDF-*aroK*-H	Plasmid is used for high intensity repression of *aroK*	This study
pCDF-*glnA*-H	Plasmid is used for high intensity repression of *glnA*	This study
pCDF-*argB*-H	Plasmid is used for high intensity repression of *argB*	This study
pCDF-*metK*-H	Plasmid is used for high intensity repression of *metK*	This study
pCDF-*metK*-M	Plasmid is used for medium intensity repression of *metK*	This study
pCDF-*metK*-L	Plasmid is used for low intensity repression of *metK*	This study
pCDF-*pro*B-H	Plasmid is used for high intensity repression of *pro*B	This study
pCDF-*pro*B-M	Plasmid is used for medium intensity repression of *pro*B	This study
pCDF-*pro*B-L	Plasmid is used for low intensity repression of *pro*B	This study
pCDF-*metK*-H-*pro*B-L	Plasmid is used for high intensity repression of *metK* and low intensity repression of *pro*B	This study
pCDF-*metK*-M-*pro*B-L	Plasmid is used for medium intensity repression of *metK* and low intensity repression of *pro*B	This study
pCDF-*metK*-L-*pro*B-L	Plasmid is used for low intensity repression of *metK* and low intensity repression of *pro*B	This study
pCDF-*fabF*-H	Plasmid is used for high intensity repression of *fabF*	This study
pCDF-*fabB*-L	Plasmid is used for low intensity repression of *fabB*	This study
pCDF-*fumC*-M	Plasmid is used for medium intensity repression of *fumC*	This study
pCDF-*sucC*-M	Plasmid is used for medium intensity repression of *sucC*	This study
pCDF-*adhE*-L	Plasmid is used for low intensity repression of *adhE*	This study
pCDF-*fabF*-H-*fabB*-L	Plasmid is used for high intensity repression of *fabF* and low intensity repression of *fabB*	This study
pCDF-*fumC*-M-*sucC*-M	Plasmid is used for medium intensity repression of *fumC* and medium intensity repression of *sucC*	This study
pCDF-*fabF*-H-*fabB*-L- *fumC*-M-*sucC*-M	Plasmid is used for high intensity repression of *fabF*, low intensity repression of *fabB,* medium intensity repression of *fumC* and medium intensity repression of *sucC*	This study
pCDF-*metK*-H-*pro*B-L-*fab*F-H-*fab*B-L-*fum*C-M-*suc*C-M	Plasmid is used for high intensity repression of *metK*, low intensity repression of *pro*B, high intensity repression of *fab*F, low intensity repression of *fab*B, medium intensity repression of *fum*C, medium intensity repression of *suc*C and low intensity repression of *adh*E	This study
pCDF-*put*A-H	Plasmid is used for high intensity repression of *put*A	This study
pCDF-*put*A-H-*pro*B-L	Plasmid is used for high intensity repression of *put*A and low intensity repression of *pro*B	This study

**Table 2:** Strains used in this study

Strain	Strain description	Source
Empty	BL21 (DE3) with pTrc-BOPAL-PA4CL, pRSF-CHS (Met) -CHI plasmid	This study
Control	Empty with pACYC-dCas9 plasmid	This study
*gfp*-sgRNA-H	Control with pCDF303-*gfp*-H plasmid	This study
*gfp*-sgRNA-M	Control with pCDF303-*gfp*-M plasmid	This study
*gfp*-sgRNA-L	Control with pCDF303-*gfp*-L plasmid	This study
Cri-*aro*K-H	Control with pCDF-*aro*K-H plasmid	This study
Cri-*gln*A-H	Control with pCDF-*gln*A-H plasmid	This study
Cri-*arg*B-H	Control with pCDF-*arg*B-Hplasmid	This study
Cri-*metK*-H	Control with pCDF-*metK*-H plasmid	This study
Cri-*metK*-M	Control with pCDF-*metK*-M plasmid	This study
Cri-*metK*-L	Control with pCDF-*metK*-L plasmid	This study
Cri-*pro*B-H	Control with pCDF-*pro*B-H plasmid	This study
Cri-*pro*B-M	Control with pCDF-*pro*B-M plasmid	This study
Cri-*pro*B-L	Control with pCDF-*pro*B-L plasmid	This study
Cri-*metK*-H-*pro*B-L	Control with pCDF- *metK*-H-*pro*B-L plasmid	This study
Cri-*metK*-M-*pro*B-L	Control with pCDF- *metK*-M-*pro*B-L plasmid	This study
Cri-*metK*-L-*pro*B-L	Control with pCDF- *metK*-L-*pro*B-L plasmid	This study
Cri-*fabF*-H	Control with pCDF-*fabF*-H plasmid	This study
Cri-*fabB*-L	Control with pCDF-*fabB*-L plasmid	This study
Cri-*fumC*-M	Control with pCDF-*fumC*-M plasmid	This study
Cri-*sucC*-M	Control with pCDF-*sucC*-M plasmid	This study
Cri-*adhE*-L	Control with pCDF-*adhE*-L plasmid	This study
Cri-*fabF*-H-*fabB*-L	Control with pCDF-*fabF*-H-*fabB*-L plasmid	This study
Cri-*fumC*-M-*sucC*-M	Control with pCDF-*fumC*-M-*sucC*-M plasmid	This study
Cri-A	Control with pCDF-*metK*-H-*pro*B-L plasmid	This study
Cri-M	Control with pCDF-*fabF*-H-*fabB*-L- *fumC*-M-*sucC*-M plasmid	This study
Cri-AM	Control with pCDF-*metK*-H-*pro*B-L-*fab*F-H-*fab*B-L-*fum*C-M-*suc*C-M plasmid	This study
Cri-*put*A-H	Control with pCDF-*put*A-H plasmid	This study
Cri-*put*A-H-*pro*B-L	Control with pCDF-*put*A-H-*pro*B-L plasmid	This study

### Construction of sgRNA-expressing plasmids

pACYC-dCas9 was constructed by amplifying dCas9 fragment from 44249-dCas9 plasmid (no. 44249) (Addgene, USA) into *Bgl*II/*Xho*I sites of pACYCDuet-1. The sgRNA chimera, which consists of five domains [a Trc-inducible promoter, a 20-nucleotide (nt) complementary region for specific DNA binding, a 42-nt dCas9-binding hairpin, a 40-nt transcription terminator derived from *Streptococcus pyogenes* and a 46-nt rrnB transcription terminator] was synthesized by Genewiz (Suzhou, China) and inserted into *Eco*RI/*Bam*HI sites of pCDF303. This resulted in the plasmid pCDF303-*gfp*-H, which was the template for PCR-based mutagenesis. Site-directed mutagenesis was performed using the overlap-extension PCR method with mutant-specific primers. Other plasmids for repression were constructed like pCDF303-*gfp*-H. But the primers are different. Oligo nucleotides used to generate sgRNA cassettes and the resultant sgRNA expression vectors are listed in Additional file 1: Tables S1 and S2. Inhibition of multiple genes is based on the ligation used isocaudomers *Bgl*II and *Bam*HI. Plasmid pCDF-*pro*B-L was cut with *Eco*RI and *Bgl*II and plasmid pCDF-*metK*-H was cut with *Eco*RI and *Bam*HI. Plasmid pCDF-*metK*-H-*pro*B-L was obtained after ligation.

### Analytic methods

To assess the levels of cinnamic acid and pinocembrin, the supernatant was extracted with an equivalent volume of ethyl acetate, vortexed, and centrifuged at 6000 rpm for 3 min at 4 °C. Then, the upper organic layer was removed and evaporated to dryness. The remaining residue was resolubilized with methanol (TEDIA, Fairfield, OH, USA). Samples were quantified by HPLC (Alltech, Deerfield, IL, USA) using an Alltech series 1500 instrument equipped with a prevail C18 reverse-phase column (5 μm, 250 × 4.6 mm; Grace, Deerfield, IL, USA) maintained at 25 °C. For detection, 0.1% acetic acid (solvent A) and acetonitrile supplemented with 0.1% acetic acid (solvent B) were applied as the mobile phases at a flow rate of 1.0 mL min^−1^. The elution was performed according to the following conditions: minute 0–1: 15% B; minutes 1–10: 15% to 40% B; minutes 10–15: 40% to 50% B; minutes 15–25: 50% to 85% B; minutes 25–30: 85% to 15% B; and minute 30–31: 15% B. Products were detected by monitoring the absorbance at 300 nm.

To quantify the malonyl-CoA and ATP concentration, the harvested cells were resuspended in 1 mL of 6% perchloric acid (ultrasonication in an ice-water bath) and neutralized with 0.3 mL of saturated potassium carbonate. The solution was centrifuged to pellet the cell debris. For detection of malonyl-CoA, 95% 0.1 M ammonium formate/5% MeOH (solvent A) and 50% 0.1 M ammonium formate/50% MeOH (solvent B) were applied as the mobile phases at a flow rate of 1.0 mL min^−1^. The elution was performed according to the following conditions: minutes 0–10: 100% A to 50% A; minutes 10–12: 50% to 100% A; minutes 12–13: 100% A. For detection of ATP and ADP, the mobile phase used was phosphate buffer consisting of 0.06 M K_2_HPO_4_ and 0.04 M KH_2_PO_4_ at pH 7.0 adjusted with 0.1 mol/L KOH and operated at a flow rate of 1 mL/min [13].

## Results

### The effect of ATP on pinocembrin production

**Fig. 2:**
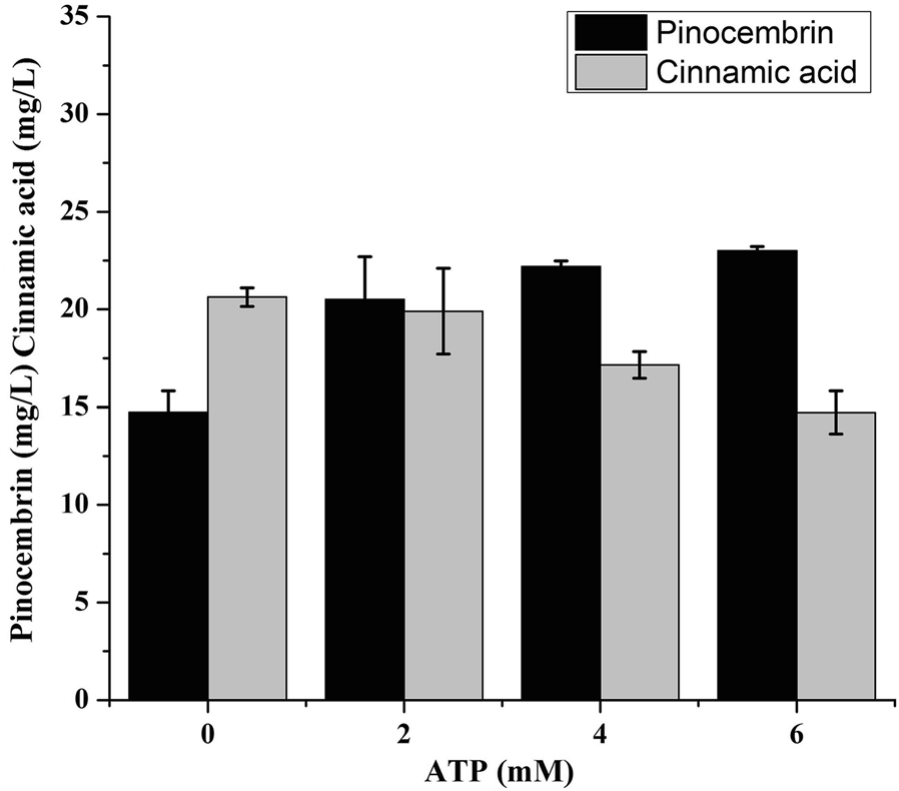
The effect of ATP additive on pinocembrin production. Different concentrations of ATP ranging from 0 to 6 mM was added in the medium

In the previous study, we constructed the pinocembrin metabolic pathway in *E. coli* BL21 (DE3) by transforming plasmids pTrc-*BO*PAL-*PA*4CL and pRSF-CHS-CHI, as a result, a pinocembrin production titer of 14.74 mg/L was achieved [6]. Using this strain as a control check (CK), to ascertain the effect of the ATP on the pinocembrin production, different concentrations of ATP ranging from 0 to 6 mM was added in the medium. As shown in Fig. 2, with an increasing ATP concentration from 0 to 6 mM, the pinocembrin concentration is increased as well. When the cultures were supplemented with 6 mM ATP, the strain exhibited the pinocembrin production of 23.00 mg/L.

### Design and construction of the CRISPRi systems for repression of the genes

According to previous research, the CRISPRi system can efficiently repress expression of targeted genes in *E. coli*, with no detectable off-target effects [20]. In the present study, we constructed our adjustable CRISPRi system in *E. coli* BL21 (DE3) and confirmed its effect by repressing the green fluorescent protein (*gfp*) expression. Three sgRNAs with different repression efficiency were designed and constructed. Three corresponding protospacer adjacent motif (PAM) was located on the 29th, 293th and 683th of *gfp* gene [19]. The closer the PAM region is to the initiation codon, the stronger the inhibition. As shown in the Additional file 1: Figure S1, compared with *gfp*-control, the fluorescence intensity of *gfp*-sgRNA-H, *gfp*-sgRNA-M and *gfp*-sgRNA-L could be decreased by 90.4%, 47.5% and 10.1% at 3 h, respectively. After 6 h, the fluorescence intensity of *gfp*-sgRNA-H, *gfp*-sgRNA-M and *gfp*-sgRNA-L could be decreased by 95.2%, 53.3% and 11.8% than that in *gfp*-control, respectively. These results indicated that CRISPRi system could be successfully repressing the expression of targeted protein in *E. coli* BL21(DE3).

### Screening of different gens for increasing ATP levels and enhancing pinocembrin production

**Fig. 3:**
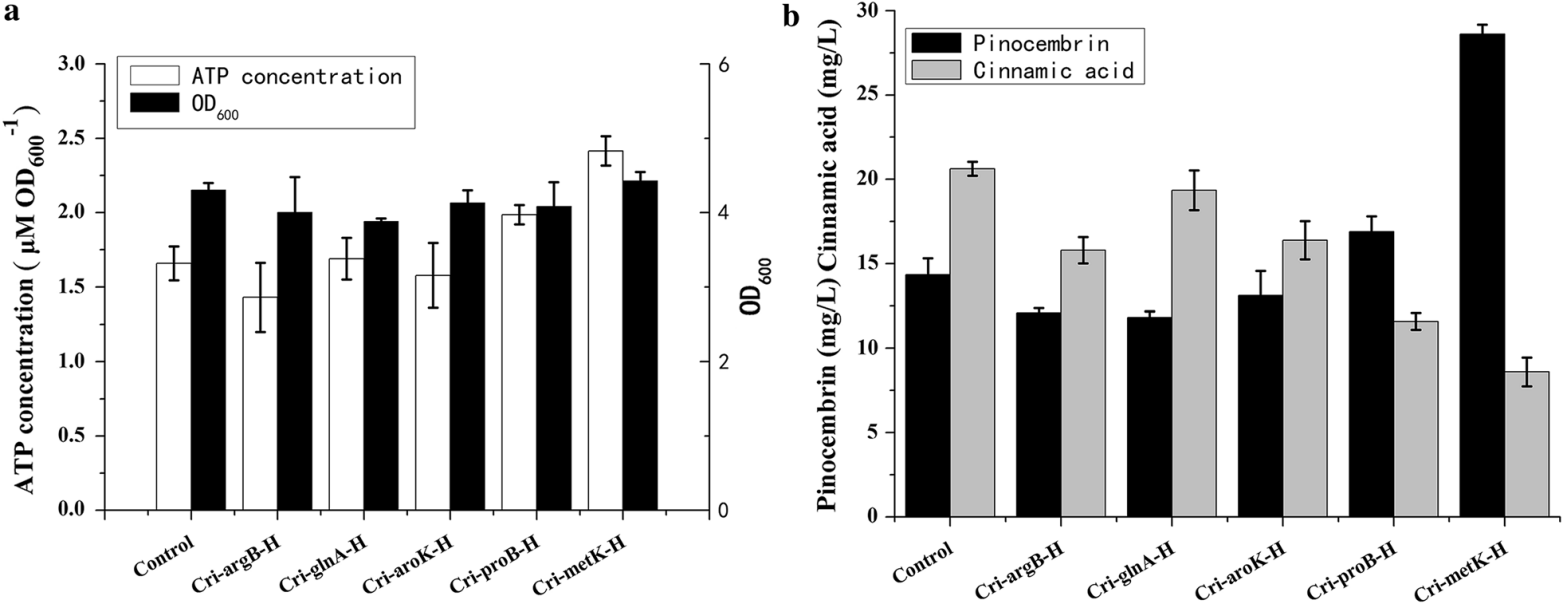
The effect of single gene high intensity repression on ATP level and product distribution. **a** The effect of single gene high intensity repression on ATP level; **b** the effect of single gene high intensity repression on pinocembrin and cinnamic acid production

In order to increase the ATP supply for pinocembrin production, we tried to suppress ATP consumption from several related pathways. The pinocembrin metabolic pathway was constructed from l-phenylalanine. Shikimate, as phenylalanine precursor, can also produce shikimate-3-phosphate through ATP-dependent reaction. So *aroK* associated with this step was selected to be repressed (Fig. 1). Further more, phosphoenolpyruvate as shikimate precursor can also generate other ATP consumption pathways such as SAM and glutamate productions. Methionine is converted to SAM by *metK*-mediated ATP-dependent reaction it follows that *metK* can be the candidate for repression. On the other hand, *proB*, *glnA* and *argB*, all involved in the synthesis of ATP-dependent downstream products of glutamate were selected as candidates for repression [13]. The CRISPRi strategy was used to fine tune the intracellular ATP level to reach the requirements for pinocembrin biosynthesis. Five high-level repression strains, Cri-*aroK*-H, Cri-*glnA*-H, Cri-*argB*-H, Cri-*proB*-H and Cri-*metK*-H were constructed to improve the ATP supply (Fig. 3). As shown in the Fig. 3a, the intracellular ATP concentration of strains Cri-*proB*-H and Cri-*metK*-H was observed to be higher than the CK while the other stains Cri-*aroK*-H, Cri-*glnA*-H and Cri-*argB*-H showed less effective on the pinocembrin production. As shown in Fig. 3b, the strains Cri-*proB*-H and Cri-*metK*-H showed positive effect on pinocembrin production, which represented 18% and 104% increase compared with CK and showed a statistically significant (p < 0.05). The cinnamic acid that produced from the strains Cri-*proB*-H and Cri-*metK*-H displayed a 38% and 54% decrease than Control. Meanwhile, the strains Cri-*aroK*-H, Cri-*glnA*-H and Cri-*argB*-H led to a negative effect on pinocembrin production compared to Control.

### Optimization of repression efficiency of proB and metK

**Fig. 4:**
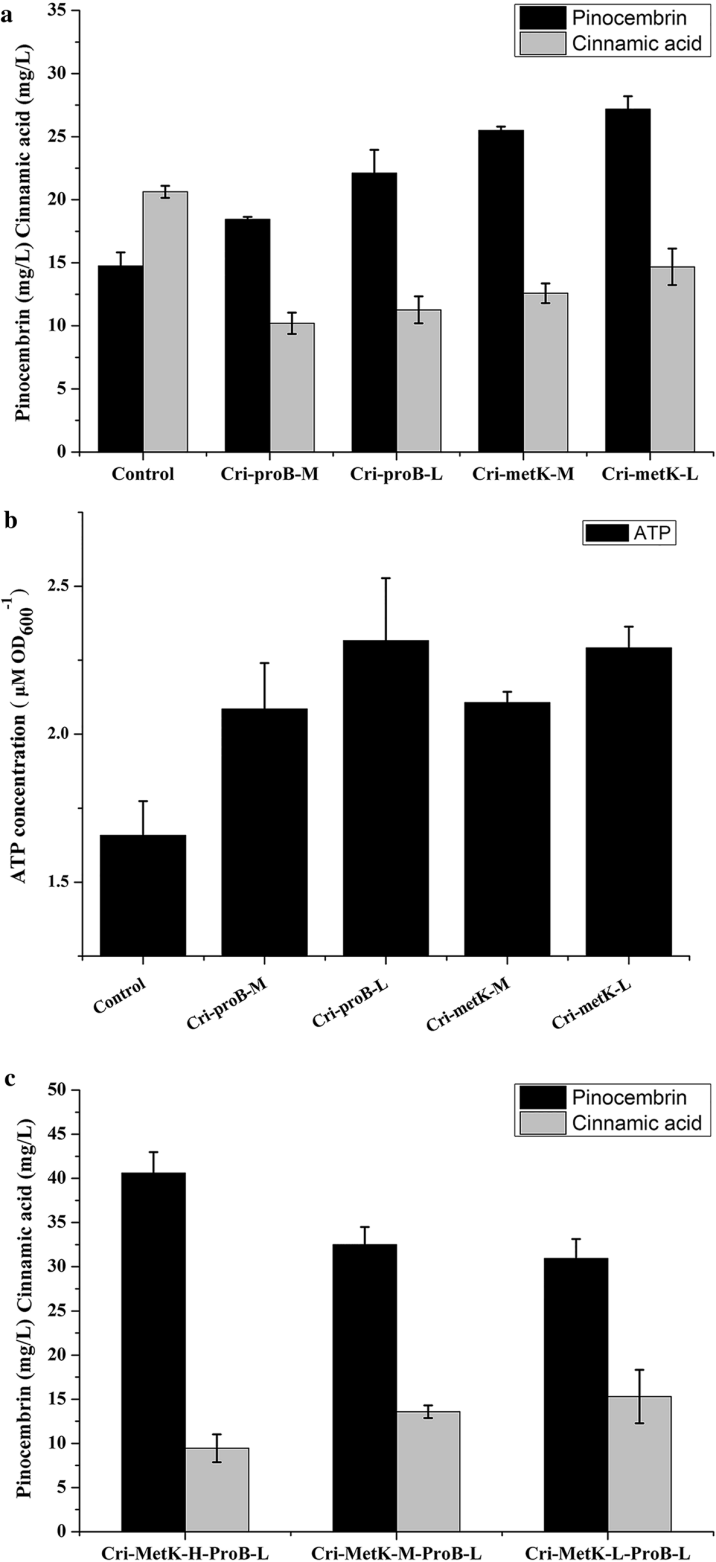
The effect of genes different intensity repression on product distribution and ATP accumulation. **a** The effect of single gene medium or low intensity repression on pinocembrin and cinnamic acid production; **b** the effect of single gene medium or low intensity repression on ATP level; **c** the effect of multiple-genes inhibition on pinocembrin and cinnamic acid production

To ascertain the effect of different repression level on pinocembrin production, different sgRNA was designed to bind the non-template DNA strand of the target gene at the initial, middle or terminal region. Therefore, corresponding PAM of *proB* was located on the 15th, 488th and 1076th of *proB* gene. Meanwhile, the PAM of *metK* was located on the 32th, 588th and 1115th of *metK* gene. As shown in Fig. 4a, an obvious increase in pinocembrin level (22.10, 25.49 and 27.18 mg/L) was observed in the strains Cri-*proB*-L, Cri-*metK*-M and Cri-*metK*-L while Cri-*proB*-M produced 16.65 mg/L pinocembrin, which is only 13% higher than that of the control strains. On the other hand, the ATP concentration of Cri-*proB*-H, Cri-*proB*-M, Cri-*proB*-L, Cri-*metK*-M and Cri-*metK*-L were increased by 20%, 26%, 40%, 27% and 38%, respectively (Fig. 4b). Particularly, with high-intensity inhibition of the *metK* gene, the ATP concentration could be increased by more than 45%. The order of conversion efficiency from high to low is Cri-*metK*-H, Cri-*metK*-M, Cri-*metK*-L, Cri-*proB*-L, Cri-*proB*-M and Cri-*proB*-H. Hence, the strains Cri-*proB*-L, Cri-*metK*-H, Cri-*metK*-M and Cri-*metK*-L were chosen as target genes for combination investigation. As shown in Fig. 4c, the strains Cri-*metK*-H-*proB*-L, Cri-*metK*-M-*proB*-L and Cri-*metK*-L-*proB*-L produced 40.59, 32.50 and 30.92 mg/L pinocembrin, respectively. The strains Cri-*metK*-H-*proB*-L was named as Cri-A for further investigation.

### Optimization of pinocembrin production by combining ATP and malonyl-CoA engineering strategy

**Fig. 5:**
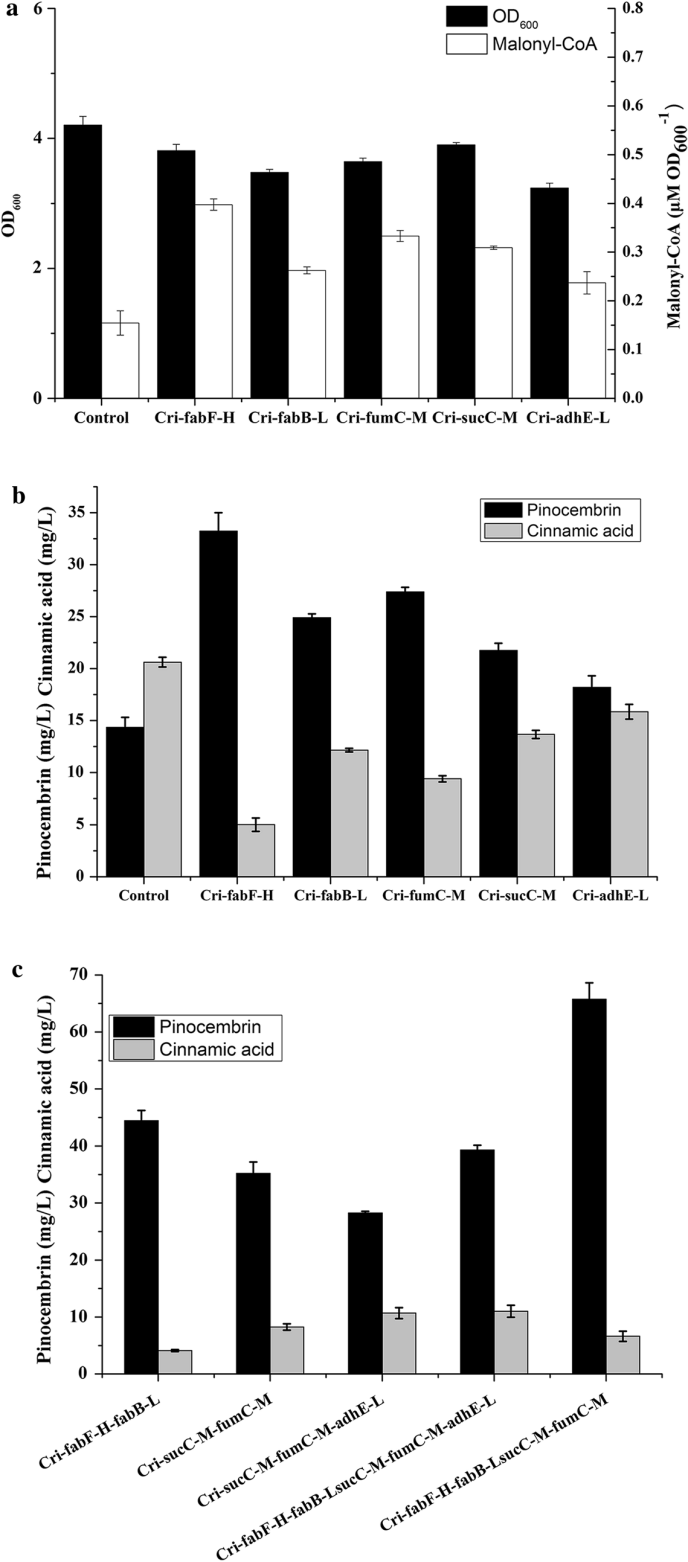
The effect of genes on malonyl-CoA level and product distribution. **a** The effect of single gene different repression on strain growth and malonyl-CoA level; **b** the effect of single gene different repression on pinocembrin and cinnamic acid production; **c** the effect of multiple-genes inhibition on pinocembrin and cinnamic acid production

As the direct precursor of pinocembrin, malonyl-CoA was proved to be significant for the pinocembrin accumulation. Acetyl-CoA is the precursor of malonyl-CoA while malonyl-CoA is the precursor of fatty acid. In order to increase the production of malonyl-CoA, some critical genes involved in relevant pathways were investigated. It has been reported that inhibition of the following genes can increase the production of malonyl-CoA: *adhE* (convert acetyl-CoA to ethyl Alcohol) [14], *fab*B and *fab*H (convert malonyl-CoA to fatty acid) [16], *fum*C and *suc*C (supplementation of TCA intermediates) [21]. To increase the malonyl-CoA level, the effect of single genetic perturbations on pinocembrin production was investigated. As shown in Fig. 5a, medium repressing efficiency of *sucC* and *fumC* decreased the final OD_600_ by 13.5% and 7.3%. Low repressing efficiency of *fabB* and *adhE* decreased the final OD_600_ by 17.4% and 23.1%. High repressing efficiency of *fabF* decreased the final OD_600_ by 9.4%. Meanwhile, the strains Cri-*fabF*-H, Cri-*fabB*-L, Cri-*fumC*-M, Cri-*sucC*-M produced an obvious increase in pinocembrin concentration (by over 50%). The strains Cri-*adhE*-L increased the final pinocembrin production to 18.19 mg/L (Fig. 5b). It was also found that the upstream genes combination (*fumC*-M-*sucC*-M) and downstream genes combination (*fabF*-H-*fabB*-L) could produce the higher pinocembrin production than the single gene. However, the genes combination (*fabF*-H-*fabB*-L-*adhE*-L) decreased the pinocembrin production than the strains Cri-*fabF*-H-*fabB*-L (data not shown). In addition, the strains Cri-*fabF*-H-*fabB*-L-*fumC*-M-*sucC*-M produced the highest pinocembrin titer (65.77 mg/L) (Fig. 5c). The strains Cri-*fabF*-H-*fabB*-L-*fumC*-M-*sucC*-M was named as Cri-M for further investigation.

**Fig. 6:**
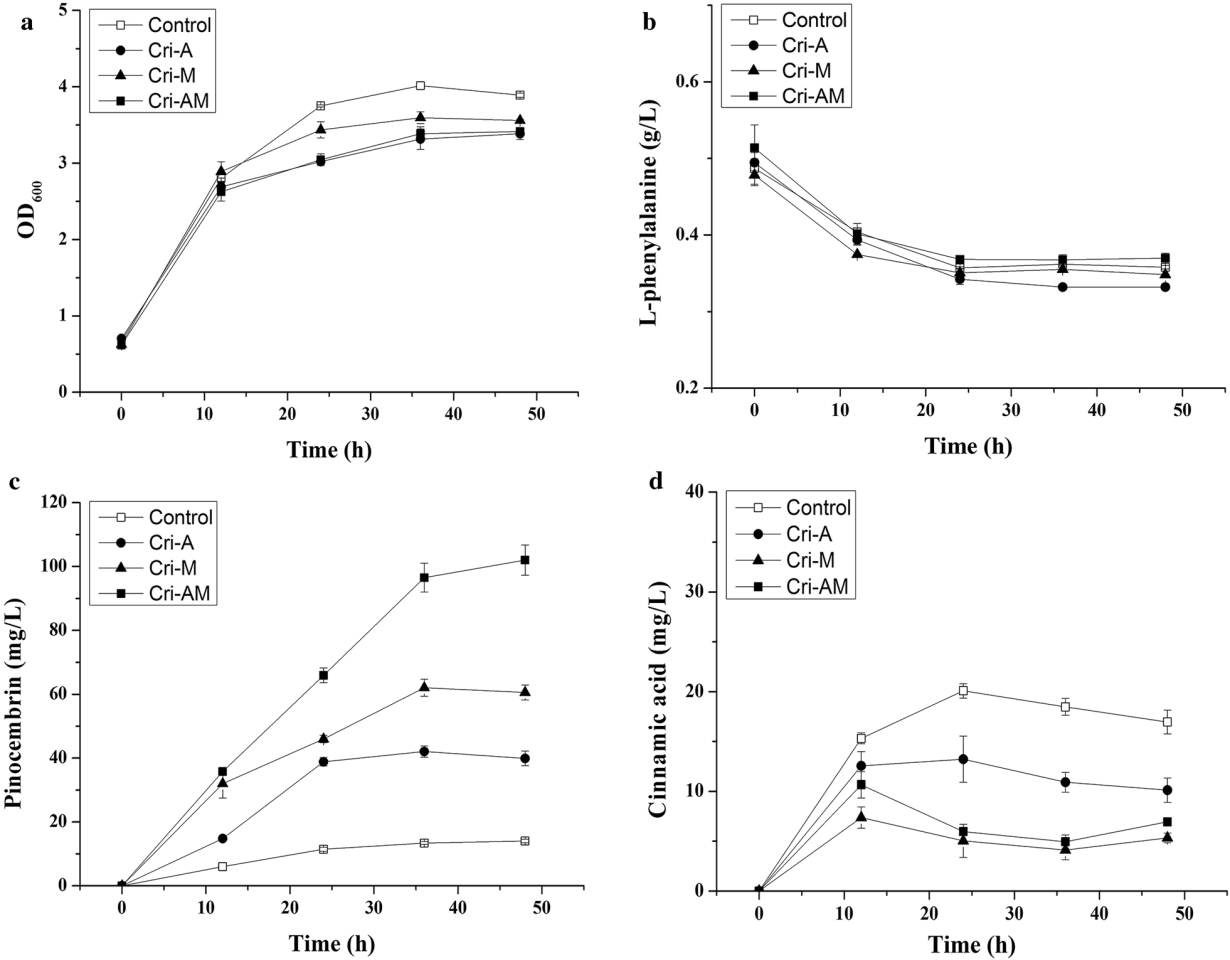
Time-course of OD600, substrate consumption and the products during 48 h fermentation by control strains, Cri-A, Cri-M and Cri-AM. **a** The effect of different strains on growth; **b** the effect of different strains on l-phenylalanine consumption; **c** the effect of different strains on pinocembrin production; **d** the effect of different strains on cinnamic acid production

**Fig. 7:**
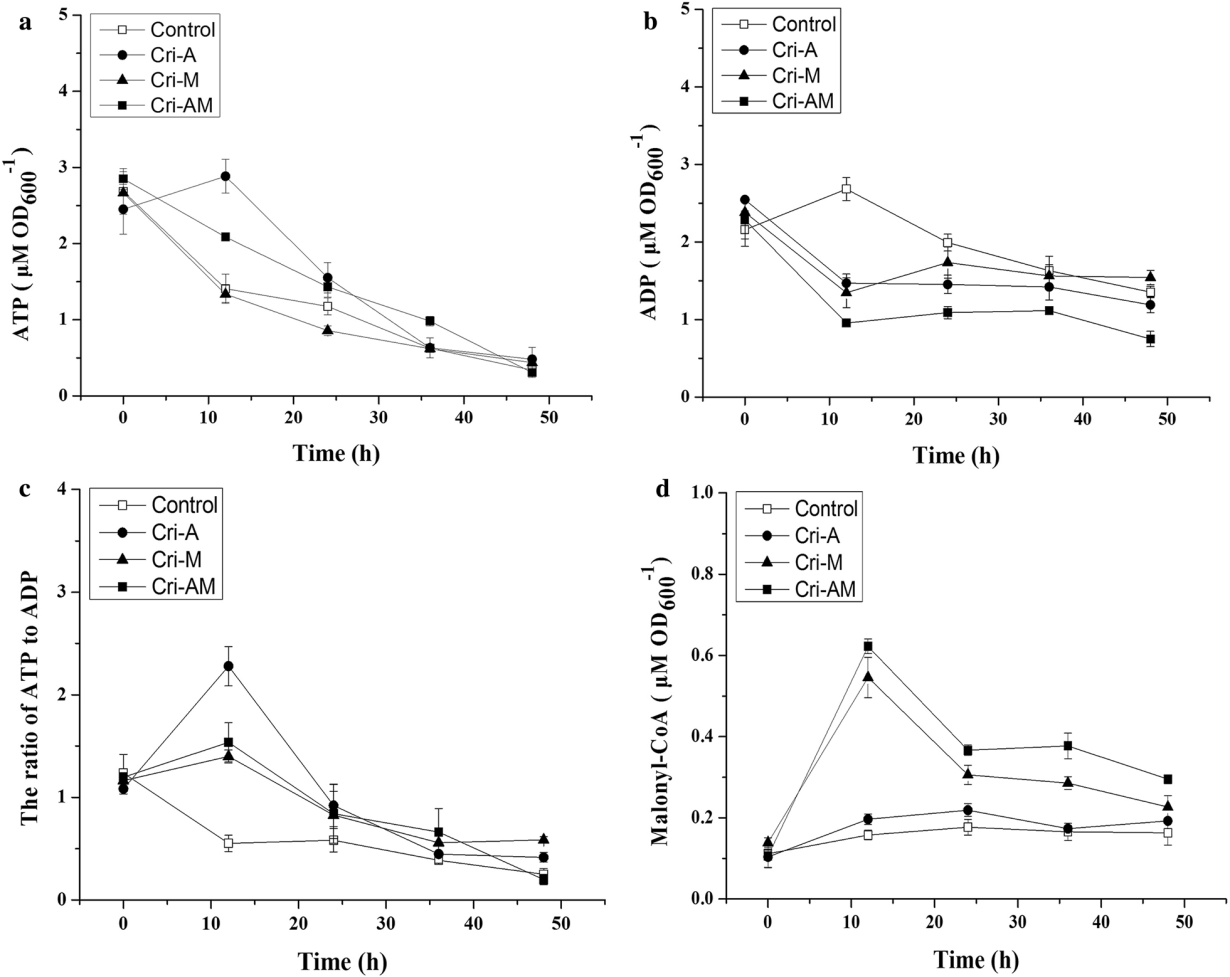
Time-course of ATP, ADP, the ratio of ATP to ADP and malonyl-CoA during 48 h fermentation by control strains, Cri-A, Cri-M and Cri-AM. **a** The effect of different strains on ATP level; **b** the effect of different strains on ADP level; **c** the effect of different strains on the ratio of ATP to ADP; **d** the effect of different strains on malonyl-CoA level

In order to further increase the pinocembrin production, the ATP engineering strategy was combined with malonyl-CoA strategy to construct the integrated strain Cri-AM. The results showed that the OD_600_ of Cri-A and Cri-AM was lower than that of the control strain (Fig. 6a), while the consumption of l-phenylalanine was almost similar (Fig. 6b). As shown in Fig. 6c, pinocembrin production by strain Cri-AM (102.02 mg/L), was 68% and 155% higher than that of strain Cri-M and Cri-A. Further, the conversion rate of intermediate cinnamic acid by strains Cri-M and Cri-AM was higher than that of strains Cri-A and control (Fig. 6d). To confirm the effect of the CRISPRi system on the ATP and malonyl-CoA concentration, the intracellular concentrations of ATP and malonyl-CoA at different cultivating time were determined. As shown in Fig. 7a, the strains Cri-A and Cri-AM produced a higher ATP level, compared with the control from 0 to 36 h. Especially at 12 h, the intracellular ATP concentration of Cri-A strain was almost double that of control. Meanwhile, the ADP level of Cri-A, Cri-M and Cri-AM were lower than that of the control strain at 0–36 h (Fig. 7b). Cri-A and Cri-M showed a higher ratio of ATP to ADP than that of the control (Fig. 7c). The strains Cri-AM and Cri-M produced a higher malonyl-CoA level, compared with the control and Cri-A from 0 to 48 h (Fig. 7d).

### Optimization of cultivation and induction conditions

**Fig. 8:**
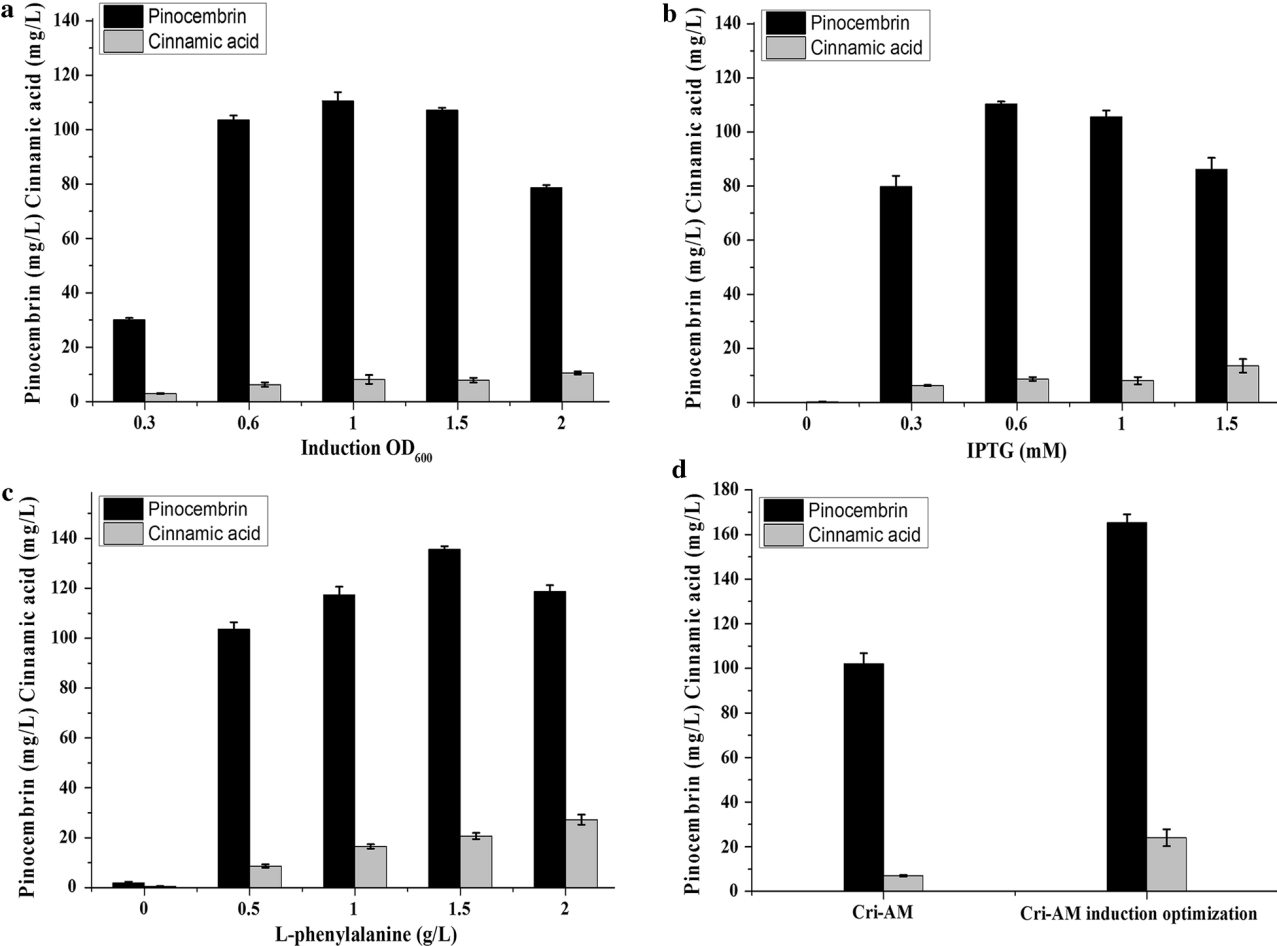
Optimization of induction conditions by Cri-AM strains. **a** Optimization of induction OD_600_; **b** optimization of IPTG concentration; **c** optimization of l-phenylalanine concentration; **d** the pinocembrin and cinnamic acid production of Cri-AM and Cri-AM after induction optimization

In order to further increase the pinocembrin production, the induction conditions and fermentation condition was optimized. First, strainCri-AM was induced at OD_600_ values of 0.3, 0.6, 1.0, 1.5 and 2.0. As seen in Fig. 8a, the production of pinocembrin was maximized at an OD_600_ of 1.0 (110.54 mg/L). Meanwhile, five concentrations of IPTG, 0, 0.3, 0.6, 1.0 and 1.5 mM, were added into the culture when the OD_600_ of Cri-AM reached around 1.0. After 48 h incubation, the pinocembrin production exhibited the highest value of 110.44 mg/L, when the concentration of IPTG was 0.6 mM (Fig. 8b). In addition, the additive of l-phenylalanine concentration was optimized. The results showed that when the l-phenylalanine addition was 1.5 g/L, pinocembrin production reached the maximum (Fig. 8c). As shown in Fig. 8d, the pinocembrin production of Cri-AM reached 165.31 mg/L under the above optimized conditions.

## Discussion

Biosynthesis of one molecule of pinocembrin requires one molecule of ATP in the pinocembrin metabolic pathway, thus, the intracellular ATP concentration might be significant for pinocembrin production. In this work, we identified a number of different genes-repression for increasing ATP and pinocembrin production in *E. coli*, and found that the repression of *metK* and *proB* based on CRISPRi system could enhance the pinocembrin production. The genes for the other enzyme tested, *glnA*, *aroK* and *argB*, had no effect. In addition, we optimized the gene repression level to increase the pinocembrin production and ATP level and further improved the pinocembrin production by multiple-gene repression of *metK* and *proB*. Combining optimization of culture, induction conditions and repression of the genes in the malonyl-CoA biosynthesis including *fabF*, *fabB*, *fumC* and *sucC*, a pinocembrin production of 11.2-fold higher than the Control were achieved.

In the central metabolism of *E. coli*, cofactors provide redox carriers for biosynthetic reactions, catabolic reactions and act as important agents in metabolism [22]. However, the low availability of cellular cofactor often impeded its utility for overproducing desired products. Although malonyl-CoA has been proved to be the limiting factor for flavonoids biosynthesis, the ATP availability in the synthetic pathway is also vital for pinocembrin production via ATP supplemented during the fermentation. In fact, the cell growth and maintenance, intracellular environment control, substrate transport and product export also require adequate supply of ATP [23]. In many cases, the optimization of the fermentation process to enhance the ATP level is an option [11]. ATP accumulation could also be elevated by gene knock-out strategies or small RNA regulation [13, 23, 24]. As CRISPRi system is advantageous over traditional gene-knockout strategies because of its ability to manipulate genes essential for growth [14], the development of CRISPRi system has offered an alternative approach to control the intracellular ATP level. The plasmid-based inducible CRISPRi system can regulate gene expression accurately at the transcriptional level in *E. coli*. Here, depending on the pET28-*gfp* plasmid locus, the non-template of *gfp* combined with dCas9-sgRNA complex, which had been successfully repressed in *E. coli* BL21(DE3) after induction by IPTG. It is found that CRISPRi system for ATP strategy did not generate growth repression in *E. coli* (data not show).

Compared with Cri-*proB*-H and Cri-*proB*-M, Cri-*proB*-L showed better effect on pinocembrin production. These finding indicate that the proline concentration might be important for *E. coli* metabolism and pinocembrin production. *MetK* was responsible for the final-product S-adenosylmethionine (SAM) in *E. coli*, while *proB* catalyze the reaction of the proline biosynthesis pathway and *putA* catalyze the reaction of the proline degradation pathway. Therefore, Cri-*putA*-H and Cri-*putA*-H-*proB*-L were constructed in order to detect whether the concentration of proline contribute to the improvement of pinocembrin production. As shown in Additional file 1: Figure S2, the strains Cri-*putA*-H decreased the pinocembrin production to 13.17 mg/L. The pinocembrin concentration in Cri-*putA*-H-*proB*-L was decreased by 30% when compared with that in Cri-*proB*-L. These results indicated that the further accumulation of proline concentration could not increase the ATP and pinocembrin production, and the repression of ATP consumption gene for proline or SAM biosynthesis was advantageous for pinocembrin production. In addition, we found that the combined strains Cri-A showed better effect than single gene interference. This demonstrated that coupling genetic modification to ATP strategy is benefit to identify the most suitable interventions. Further, the recombinant strains Cri-A and Cri-AM produced higher ATP, ATP/ADP ratio and pinocembrin production, compared with the control. Therefore, these results indicate that the ATP concentration and the ATP/ADP ratio are associated with the pinocembrin production, and high level of ATP was benefit for pinocembrin biosynthesis. Moreover, the metabolism of malonyl-CoA, as with ATP, is highly connected with the metabolic network in *E. coli*. Due to its direct association with cell growth and synthesis of phospholipids and fatty acids, the intracellular availability of malonyl-CoA is limited [21]. Compared with ATP level, more malonyl-CoA was necessary for strains to achieve a high yield of pinocembrin. By coupling malonyl-CoA genetic modification to ATP strategy, it was found that, the combined effects obviously improved the malonyl-CoA level and pinocembrin production from 0 to 48 h. These results indicate that the malonyl-CoA concentration is associated with the pinocembrin production. The present work demonstrates a way to approach the efficient biosynthesis of pinocembrin via ATP level strengthen and induction conditions optimization in *E. coli.*

## Conclusions

In this work, we found that the addition of ATP contributes to the synthesis of pinocembrin. Five ATP-related genes were screened using the CRISPRi system, and inhibition of *proB* and *metK* was found to contribute to the accumulation of ATP and pinocembrin. On this basis, the repression intensity of *proB* and *metK* were optimized, and the results showed that low intensity repression of *proB* or high intensity repression of *metK* could better increase the production of pinocembrin. The effects of low intensity repression of *proB* and high intensity repression of *metK* on the synthesis of pinocembrin were investigated. The results of the study showed that the recombinant strain Cri-A produced a higher yield of pinocembrin (40.59 mg/L). In addition, ATP strategy coupled with the malonyl-CoA engineering and optimization of culture and induction condition, the production of pinocembrin by the recombinant strain increased by more than 7 times (102.02 mg/L) compared to the control.

## Additional file

**Additional file 1: Table S1.** Primers and plasmids for single gene repression. **Table S2.** Primers and plasmids for repression of genes involved in malonyl-CoA consumption. **Figure S1.** The effect of different sgRNA on fluoresceneon 3h and 6h. **Figure S2.** The effect of *putA* on pinocembrin production.
