# Supplementary material for: Regulation of ATP levels in Escherichia coli using CRISPR interference for enhanced pinocembrin production
Source: Microb Cell Fact. 2018 Sep 18;17:147. doi: 10.1186/s12934-018-0995-7 (PMC6142380; doi:10.1186/s12934-018-0995-7)

**Journal name:** Microbial Cell Fsctories

**Manuscript Title:** Regulation of ATP levels in Escherichia coli using CRISPR interference for enhanced pinocembrin production

**The name(s) of the author(s):** Sha Tao^1^, Ying Qian^1^, Xin Wang^1^, Weijia Cao^1^, Weichao Ma^1^, Kequan Chen^1*^, Pingkai Ouyang^1^

**The affiliation(s) and address (es) of the author(s)**: 1. State Key Laboratory of Materials-Oriented Chemical Engineering, College of Biotechnology and Pharmaceutical Engineering, Nanjing Tech University, Nanjing 211816, Jiangsu, China

**The e-mail address and telephone numbers of the corresponding author:** Email: [kqchen@njtech.edu.cn](mailto:kqchen@njtech.edu.cn)

TEL.: +86-138-141-80652

**Table. S1** Primers and plasmids for single gene repression

| Target Gene | Oligo | Sequence（5’-3’） | Plasmid |
| --- | --- | --- | --- |
| *pro*B | *pro*B-H-F | CACCAGCGTCGTTTTAGAGCTAGAAATAGCAAGTTAAAATAAGGC | pCDF-*pro*B-H |
|  | *pro*B-H-R | GTAAAACTCGGAAATTGTTATCCGCTCACAATTCCAC |  |
|  | *pro*B-M-F | AACAGTTTATCGTTTTAGAGCTAGAAATAGCAAGTTAAAATAAGGC | pCDF-*pro*B-M |
|  | *pro*B-M-R | ATAAACTGTTGCTGCTGACCGAAATTGTTATCCGCTCACAATTCCAC |  |
|  | *pro*B-L-F | TCACGGTGAACGTTTTAGAGCTAGAAATAGCAAGTTAAAATAAGGC | pCDF-*pro*B-L |
|  | *pro*B-L-R | TTCACCGTGATGACATGATTGAAATTGTTATCCGCTCACAATTCCAC |  |
| *aro*K | *aro*K-F | AATAGTGCTTTTTCGTTTTAGAGCTAGAAATAGCAAGTTAAAATAAGGC | pCDF-*aro*K-H |
|  | *aro*K-R | GCACTATTGGGCGCGAAATTGTTATCCGCTCACAATTCCAC |  |
| *arg*B | *arg*B-F | TGATAATTAAGTTTTAGAGCTAGAAATAGCAAGTTAAAATAAGGC | pCDF-*arg*B-H |
|  | *arg*B-R | AACTGGGCGGGAAATTGTTATCCGCTCACAATTCCAC |  |
| *gln*A | *gln*A-F | ACGTGTTCAGGTTTTAGAGCTAGAAATAGCAAGTTAAAATAAGGC | pCDF-*gln*A-H |
|  | *gln*A-R | ACTGACGATGGAAATTGTTATCCGCTCACAATTCCAC |  |
| *metK* | *metK*-H-F | CCTTCAGAGAGTTTTAGAGCTAGAAATAGCAAGTTAAAATAAGGC | pCDF-*metK*-H |
|  | *metK*-H-R | GCATCCTGACGAAATTGTTATCCGCTCACAATTCCAC |  |
|  | *metK*-M-F | GCAGCGATTTCGTTTTAGAGCTAGAAATAGCAAGTTAAAATAAGGC | pCDF-*metK*-M |
|  | *metK*-M-R | GAAATCGCTGCAAGAAGCGGGAAATTGTTATCCGCTCACAATTCCAC |  |
|  | *metK*-L-F | GCTGCGCTTTGTGTTTTAGAGCTAGAAATAGCAAGTTAAAATAAGGC | pCDF-*metK*-L |
|  | *metK*-L-R | ACAAAGCGCAGCTGCTGCGCGAAATTGTTATCCGCTCACAATTCCAC |  |

**Table. S2** Primers and plasmids for repression of genes involved in malonyl-CoA consumption

| Oligo | Sequence（5’-3’） | | Plasmid |  |
| --- | --- | --- | --- | --- |
| *fabH*-F | GTCCGCACTTGTTCGTTTTAGAGCTAGAAATAGCAAGTTAAAATAAGGC | | pCDF-*fabH*-H |  |
| *fabH*-R | GAACAAGTGCGGACAAACGCGAAATTGTTATCCGCTCACAATTCCAC | |  |  |
| *fabB*-F | GCGCATTACCAGCGGTTTTAGAGCTAGAAATAGCAAGTTAAAATAAGGC | | pCDF-*fabB*-L |  |
| *fabB*-R | CGCTGGTAATGCGCAAGCTGGAAATTGTTATCCGCTCACAATTCCAC | |  |  |
| *sucC-F* | | GCCCATGAAGATTTGTTTTAGAGCTAGAAATAGCAAGTTAAAATAAGGC | pCDF-*suc*C-M | |
| *sucC-R* | | AAAACAAATCTTCATGGGCCTGGCGGAAATTGTTATCCGCTCACAATTCCAC |  |  |
| *fumC-F* | | CGCATACTCCGGATGTTTTAGAGCTAGAAATAGCAAGTTAAAATAAGGC | pCDF-*fum*C-M | |
| *fumC-R* | | AAAACAAATCTTCATGGGCCTGGCGGAAATTGTTATCCGCTCACAATTCCAC |  |  |
| *adhE*-F | | GCTCGGAGATCAGCGTTTTAGAGCTAGAAATAGCAAGTTAAAATAAGGC | pCDF-*adh*E-L | |
| *adhE*-R | | CTAAAACGCTGATCTCCGAGCTGAAACGAAATTGTTATCCGCTCACAATTCCAC |  |  |

**Figure S1** The effect of different sgRNA on fluoresceneon 3h and 6h


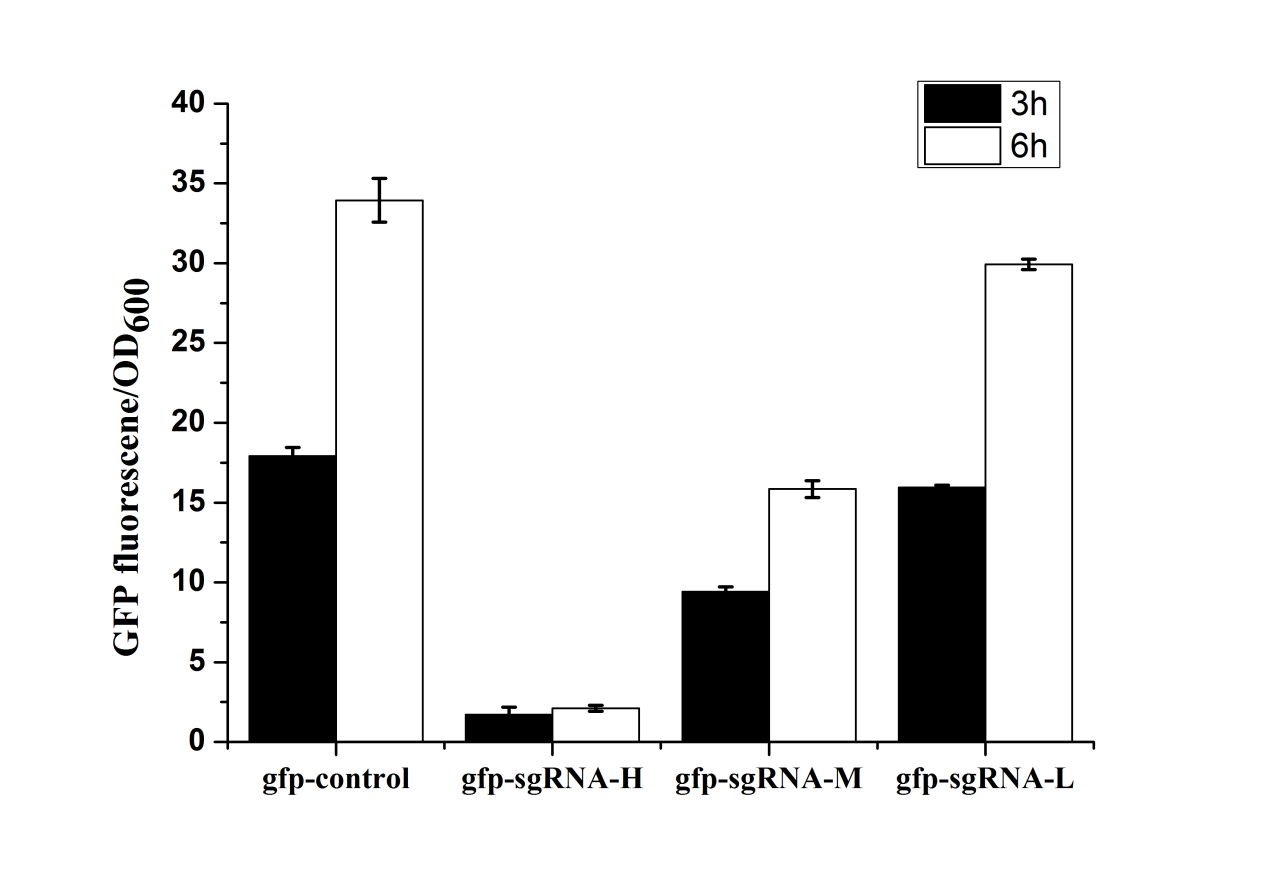


**Figure S2** The effect of *putA* on pinocembrin production


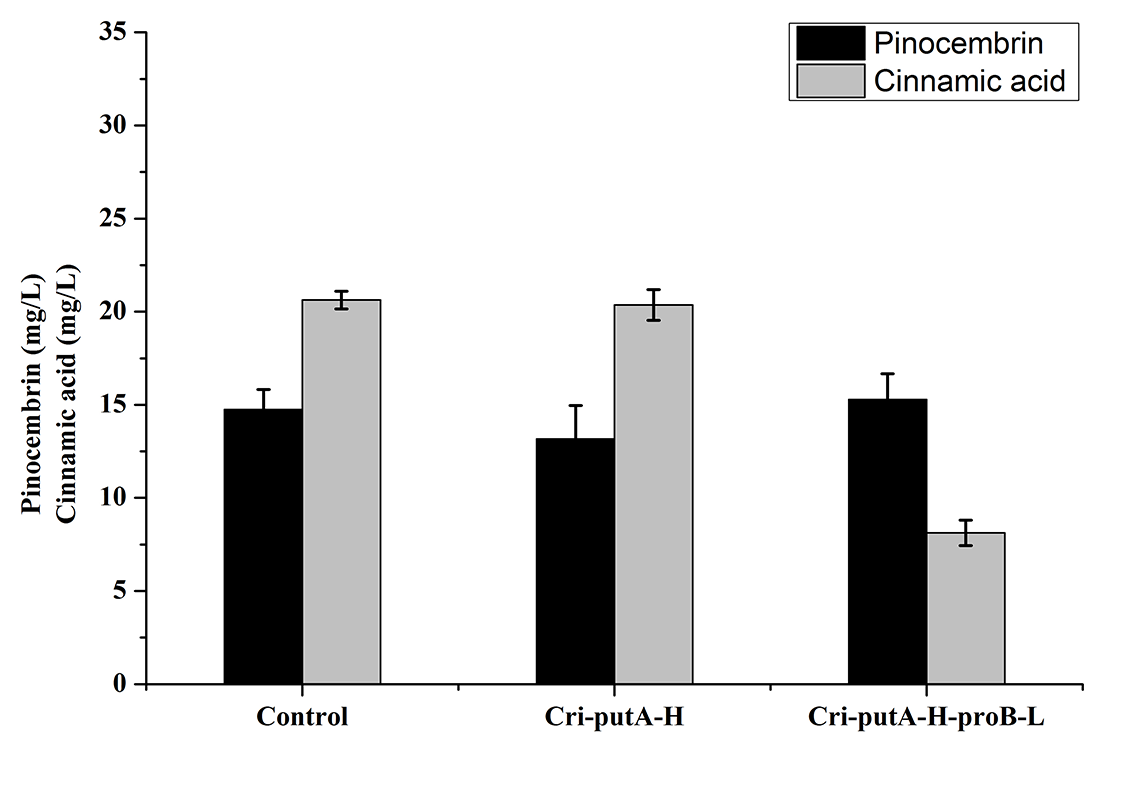

Supplement: Supplementary file 1 — Additional file 1: Table S1. Primers and plasmids for single gene repression. Table S2. Primers and plasmids for repression of genes involved in malonyl-CoA consumption. Figure S1. The effect of different sgRNA on fluoresceneon 3h and 6h. Figure S2. The effect of putA on pinocembrin production. [file 12934_2018_995_MOESM1_ESM.docx]
